# Realize, Analyze, Engage (RAE): A Digital Tool to Support Recovery from Substance Use Disorder

**DOI:** 10.20900/jpbs.20210002

**Published:** 2021-02-24

**Authors:** Stephanie Carreiro, Melissa Taylor, Sloke Shrestha, Megan Reinhardt, Nicole Gilbertson, Premananda Indic

**Affiliations:** 1 Division of Medical Toxicology, Department of Emergency Medicine, University of Massachusetts Medical School, Worcester, MA 01655, USA; 2 Department of Electrical Engineering, The University of Texas at Tyler, Tyler, TX 75799, USA; 3 RAE Health, Bristol, ME 04539, USA

**Keywords:** substance use disorder, stress, craving, mHealth, wearable, digital health, digital diagnostics, digital therapeutics

## Abstract

**Background:**

Substance use disorders are a highly prevalent group of chronic diseases with devastating individual and public health consequences. Current treatment strategies suffer from high rates of relapse, or return to drug use, and novel solutions are desperately needed. Realize Analyze Engage (RAE) is a digital, mHealth intervention that focusses on real time, objective detection of high-risk events (stress and drug craving) to deploy just-in-time supportive interventions. The present study aims to (1) evaluate the accuracy and usability of the RAE system and (2) evaluate the impact of RAE on patient centered outcomes.

**Methods:**

The first phase of the study will be an observational trial of *N* = 50 participants in outpatient treatment for SUD using the RAE system for 30 days. Accuracy of craving and stress detection algorithms will be evaluated, and usability of RAE will be explored via semi-structured interviews with participants and focus groups with SUD treatment clinicians. The second phase of the study will be a randomized controlled trial of RAE vs usual care to evaluate rates of return to use, retention in treatment, and quality of life.

**Anticipated findings and future directions:**

The RAE platform is a potentially powerful tool to de-escalate stress and craving outside of the clinical milieu, and to connect with a support system needed most. RAE also aims to provide clinicians with actionable insight to understand patients’ level of risk, and contextual clues for their triggers in order to provide more personalized recovery support.

## INTRODUCTION

Substance use disorders (SUDs) compose a group of chronic relapsing diseases, characterized by recurrent use of substances (including alcohol and recreational drugs) leading to negative physical, social, legal and financial consequences [1]. In 2018, 20.3 million Americans suffered from a SUD [2], resulting in staggering healthcare costs. As with other chronic diseases, SUDs can be treated. However the efficacy of traditional treatment options, which largely focus on a combination of behavioral and pharmacologic therapies, is limited by the relapsing nature of the disease. It is estimated that an average of 40–60% of individuals in recovery will have an episode of return to drug use (or relapse), with rates for some SUDs reported as high as 91% [3]. Although return to use is an acknowledged aspect of the recovery process, it represents a dangerous and potentially lethal situation: for example, individuals who return to opioid use after a period of abstinence are at an increased risk of overdose due to their decreased tolerance. Though treatment programs instill techniques for recognizing and avoiding drug use triggers, these may be difficult to apply in real world settings when patients are outside the protected clinical environment.

Stress is a documented risk factor for return to drug use. For individuals with SUD, stress can mimic a withdrawal like state and can lead to poor decision making [4,5]. Stress has been positively correlated to craving for opioids, cocaine and tobacco, and to drug relapse in individuals in treatment for SUD [6–9]. Stress is therefore indicative of high-risk periods in recovery, and the identification of stress may predict risk of relapse in susceptible individuals. Craving, or the subjective experience of wanting to use a drug, is another important behavioral phenomenon to consider in recovery—it is included in the DSM-IV diagnosis of SUD, is a strong predictor of return to use, and carries an interesting, likely bidirectional, relationship to stress [5,10–12]. That is, craving may be precipitated by or induce stress, and the two states share some physiological characteristics. Both stress and craving manifest physiologic changes that can be detected as digital biomarkers by wearable biosensors and can reveal prime opportunities for real time adaptive intervention [13]. Addressing stress via structured interventions, such as mindfulness exercises, has demonstrated significant positive effects on treatment success [14,15]. Similarly, overall mindfulness has been inversely correlated with craving, and mindfulness based interventions have been shown to decrease drug craving in multiple trials [16–18].

Current treatment protocols largely fail to tap into the potential of mHealth as a powerful tool to combat addiction. Wearable sensors are noninvasive devices that are capable of detecting the biomarkers associated with stress and craving. Many of these wearable sensors are commercially available smart watches and are well-received by patients [19–21]. Wearable sensors are already under investigation for the detection and treatment of a number of clinical disorders, such as epilepsy, PTSD, suicidality, cardiac arrythmias, stress and drug abuse [22–31]. mHealth capitalizes on the burgeoning industry of mobile applications (apps) and wearable sensors to connect clinicians and patients, to understand behaviors and experiences in natural environments, and to offer real time interventions.

Early work efforts in the development of mHealth based tools for SUDs have encountered challenges, including limited accuracy of digital biomarker detection, acceptability of digital health tools by consumers, privacy issues regarding continuous monitoring and data streaming, lack of standard methods for analysis, and implementation barriers. Despite barriers however, mobile health technologies are rapidly becoming more sophisticated, and are showing increasing promise in treatment paradigms. Prior studies have demonstrated the usability, efficacy and acceptability of technology-based interventions to support individuals in recovery from SUD [13,32–35]. For example, Gustafson et al. have demonstrated the efficacy of a mobile phone app to decrease risky drinking days and increase likelihood of abstinence in alcohol use disorder (AUD) [33]. The mobile app (ACHESS) uses cravings based on ecological momentary assessment (EMA) and geolocations to trigger personalized feedback and support. In marijuana users, Monney et al. found the Stop-cannabis app, which delivers personalized messages based on self-reported marijuana use, demonstrated high perceived usefulness and high acceptability [35]. Although mobile apps are common, few mHealth interventions leverage continuous physiologic sensors to trigger interventions. One such system, described by Leonard et al., also used a mobile app (Mind the Moment) for AUD combining EMA with physiologic data (electrodermal activity from a wearable sensor) to trigger their personalized messages and education [34]. In a pilot study of young adults, acceptability and perceived helpfulness of the app were high. However to our knowledge no intervention exists to combine objective detection of multiple markers of risk with real time support in addition to de-escalation tools.

An urgent need exists for treatment options that immediately identify periods of high risk, predict relapse, and intervene in real time- before relapse occurs. We propose Realize Analyze Engage (RAE) as a solution to bridge this gap in treatment. RAE is the first mHealth intervention to our knowledge to utilize a wearable sensor for continuous monitoring of stress and craving detection with real-time mobile interventions. RAE provides an approach to substance use treatment by combining digital detection of high-risk stress periods and drug use (to help users REALIZE and ANALZYE these to critical states), with interventions, support and trend reporting (to help users ENGAGE in their treatment program). The ability of clinicians to tailor interventions for individuals in recovery based on monitoring of physiologic parameters has the potential to prevent relapse, improve rates of sustained recovery, and save lives compared to current treatment paradigms. The present study aims to 1) evaluate the accuracy of stress and craving detection within RAE, and to assess usability of RAE from the patient and clinician standpoint and 2) evaluate the impact of RAE on outcomes of return to drug use, retention in treatment and quality of life.

## RAE SYSTEM ARCHITECTURE

**Figure 1.**
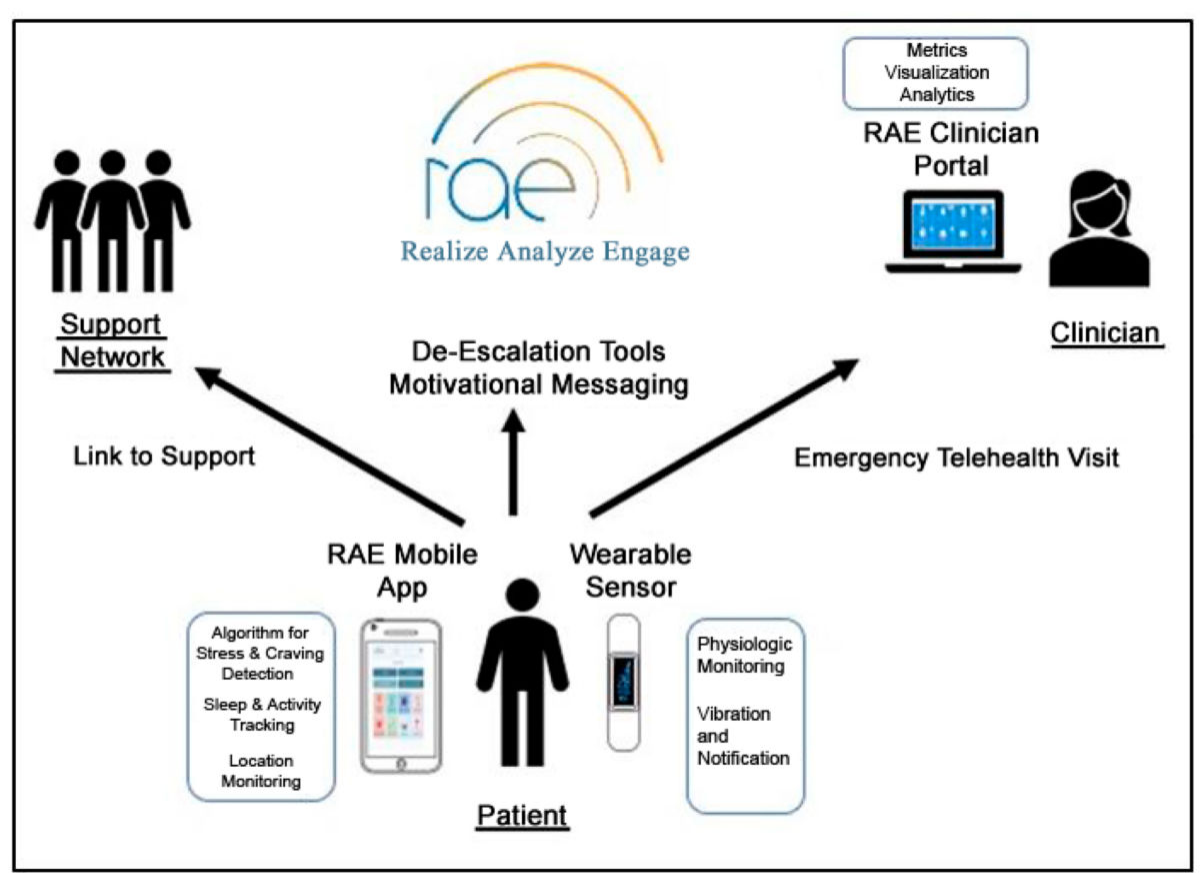
RAE System Architecture.

The RAE system (Figure 1) is an innovative mHealth intervention for individuals in recovery for SUD that combines objective metrics with digital therapeutics to promote sobriety and self-efficacy through behavior modification. The RAE system consists of (1) a wearable device that continuously measures physiology (2) an interactive mobile app that offers dialectical behavior therapy (DBT)-based interventions to promoted mindfulness upon stress or craving detection in wearable sensor data, and (3) a clinical portal to aggregate data, provide monitoring capabilities and provide actionable insight to treatment providers. The RAE systems (app and clinical portal) are HIPAA, HITECH Act and CFR 42 part 2 compliant.

The RAE system was developed by a team of physicians, engineers, web and app developers based on several years of preliminary work. The patented algorithm for the detection of stress and craving is based on a pilot study in 30 individuals in recovery from SUD conducted at two outpatient treatment centers in New England [13]. In the pilot study participants wore a wore a wrist-mounted wearable sensor that measured electrodermal activity (skin conductance), tri-axial accelerometry (i.e., locomotion in three directions), heart rate, heart rate variability, and skin temperature for four days, and self-reported episodes of stress and craving. A total of 25 features were obtained from the five sensor datastreams. Forty-minute segments of data surrounding events (20 min pre- and 20 min post- self-reported stress or craving), and were analyzed using a 5-min siding window operation. Based on the features using shape and scale derived from accelerometer and heart rate data from our preliminary work, we are able to differentiate stress from non-stress condition with an accuracy of 74.5%, and cravings from no-cravings with an accuracy of 75.7% using Fine Gaussian Support Vector Machine model.

The workflow, interaction components and aesthetic of RAE were iteratively refined based on input from target end users—both patients in SUD treatment and providers. Specifically, semi-structured interviews were conducted with the 30 participants from the pilot study to assess acceptability, barriers and facilitators to sensor-based monitoring. Participants were also allowed to demo the app and provide feedback (although the working app was not available to use it he initial pilot). Two focus groups with treatment staff (*N* = 6 in each group) were conducted to assess needs, potential integrations into workflow, and was used to refine and develop the app and clinical portal.

### The Wearable Sensor

**Figure 2.**
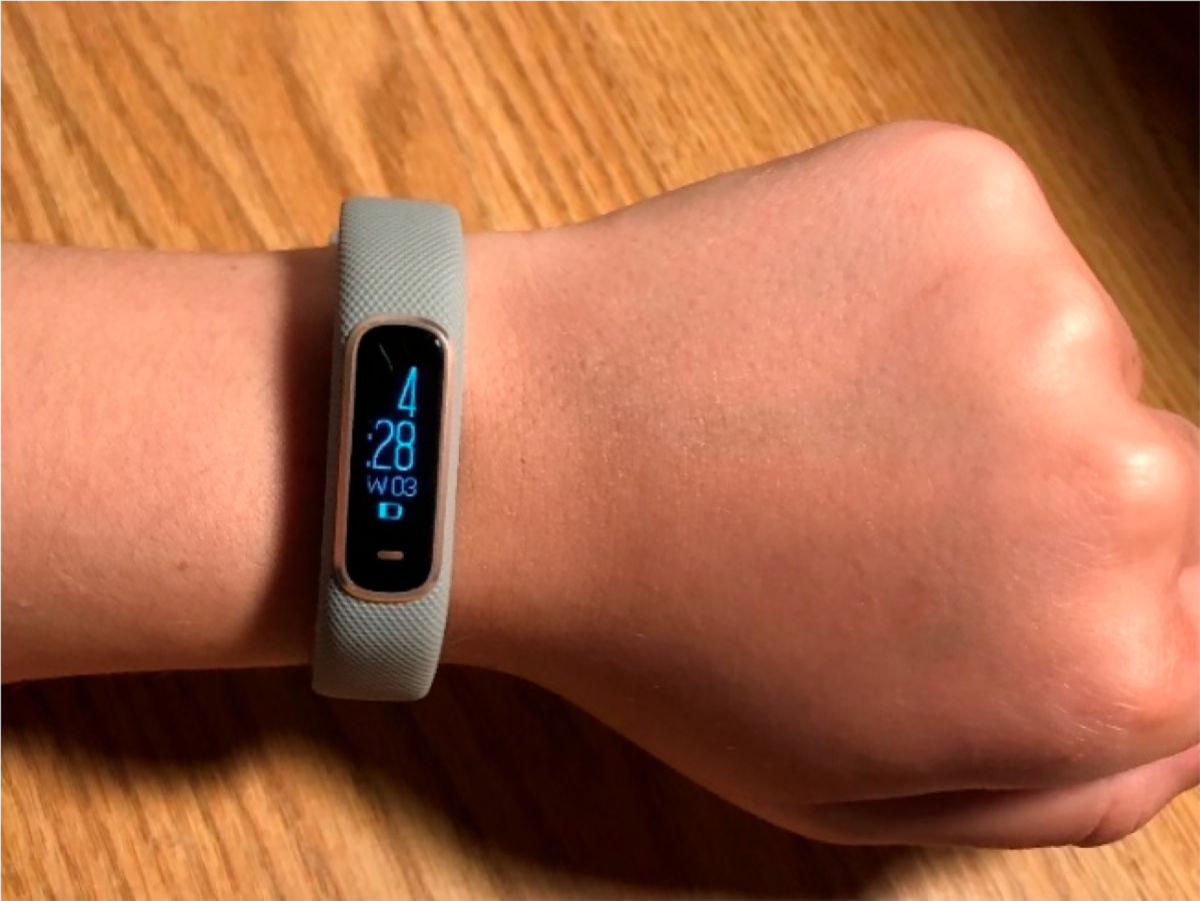
Garmin Vivosmart 4.

The first component of the RAE system is a wrist worn wearable sensor to continuously detect physiologic data, which are streamed continuously via Bluetooth connection to the RAE app. The current version of the app pairs with the Garmin Vivosmart 4 (Figure 2, Garmin, Olathe, KS, USA). The Vivosmart 4 is equipped with a barometric altimeter, tri-axial accelerometer, heart rate monitor, and pulse oxygenation sensor. The Vivosmart 4 has a touch screen interface, has up to 7 days of battery life, is waterproof, and also functions as a standard fitness tracker, making it appealing to RAE users. Future iterations of the RAE app will be device agnostic and able to connect to other popular commercially available smartwatches that include the minimum required sensor components.

### RAE Mobile Phone Application

**Figure 3.**
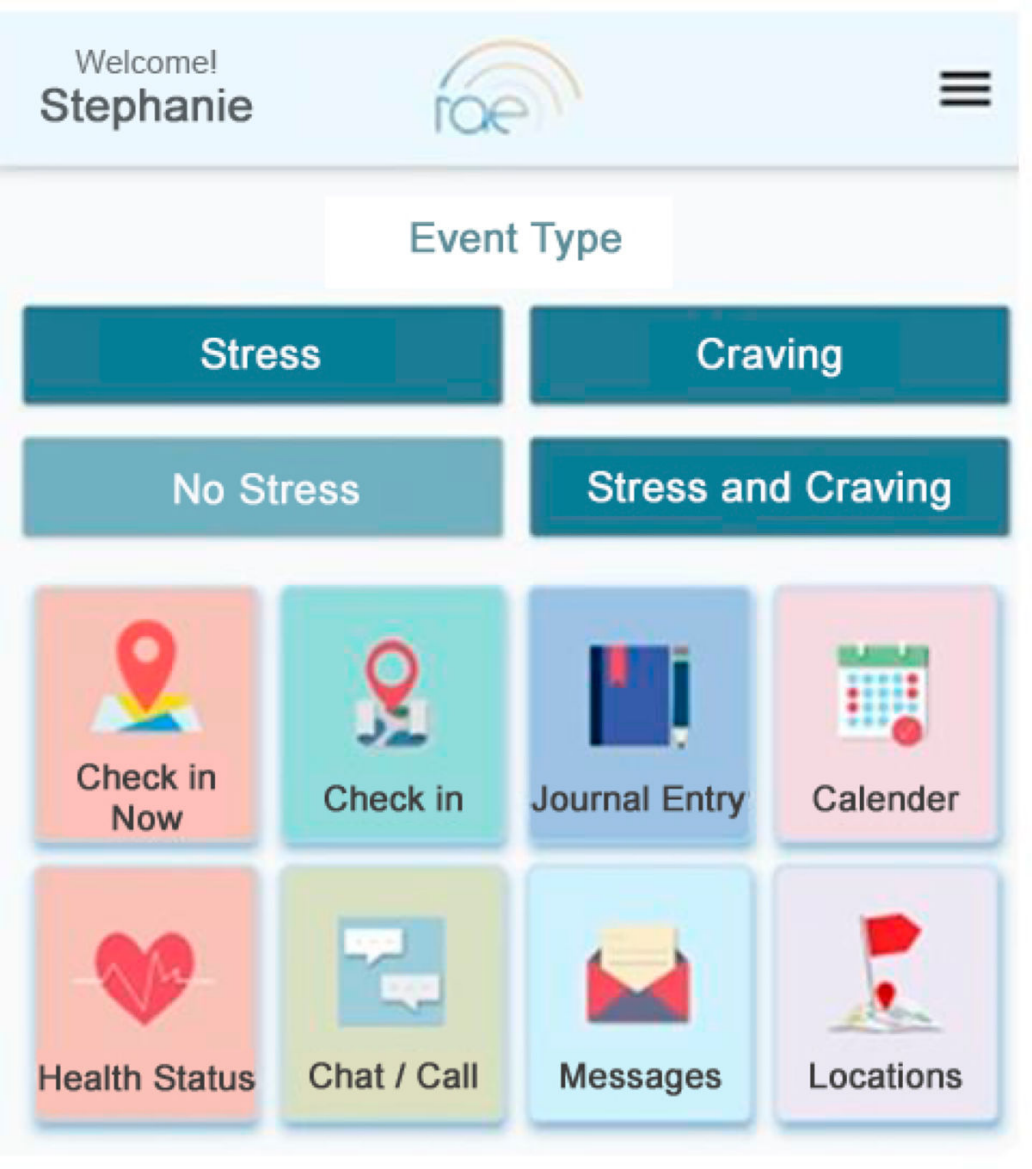
RAE Mobile App.

The RAE mobile application (Figure 3) is available for Android and iOS. The app includes interactive tools that are prompted when an event of interest is detected based on wearable sensor or phone sensor data (i.e., stress, craving, or physical proximity to a location). Tools can also be manually initiated by the user at their discretion. Features of the RAE app include de-escalation tools, geolocation based monotiling tools, and general health monitoring tools.

#### De-escalation tools

The RAE app offers a journaling tool with multiple DBT based prompts. These are automatically requested once an event has been detected, but can also be accessed on demand. Journal prompts focus on current circumstances and emotions, the users perceptions of them and their intensity rating. Journal entries are intended to promote mindfulness and to serve as a data collection tool understand patterns and triggers. Users also have access to a brief, guided breathing tool to help de-escalate perceived stress. Finally, upon detection of stress or craving based on our patented algorithm, the app will offer the user emergency help in the form of an audio or video call to one of their pre-defined support persons. These may be personal contacts or providers from their treatment program (e.g., case managers, peer recovery coaches). If the selected contact is a healthcare provider in a telehealth enabled treatment program who is “available” in the portal, an option to request an emergency telehealth visit is presented.

#### Check in and location monitoring tools

This feature uses geolocation to monitor both desirable locations to demonstrate accountability and build trust during recovery, and undesirable locations to provide support. Manual check-ins can be initiated by the user to demonstrate presence at location important to treatment success (for example checking in at their place of employment on time, or checking in to a 12-step group meeting). Areas considered high risk (e.g., a familiar liquor store) can be pre-programmed in the app by a clinician, and will trigger an alert if the patient’s phone comes within at least 100 meters of the tagged location. When either of these features are engaged, the geocoded location is then logged in the app. Both prompt supportive messages that align with treatment goals. For example a check in at a meeting will prompt praise for continuing to meet treatment goals, and a detection in a risky location will prompt a personalized motivational message with a reminder engage their support network if needed. Geocoded location for any detected or manually entered stress or craving event it also logged in the RAE system.

#### General health monitoring tools

Quality and duration of sleep and physical activity as detected by the wearable are also recorded by the app. Events, activity, sleep, and location are plotted over time in the calendar with color coding schemes to provide insight into temporal, situational and geographic relationships.

### The RAE Clinical Portal

**Figure 4.**
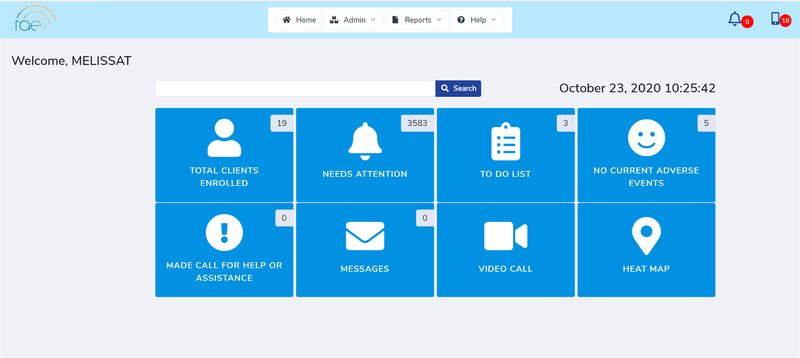
RAE Clinical Portal Home Screen.

The RAE Clinical Portal (Figure 4) will be accessible only by treatment providers and will capture all data from the user. The portal maintains historical data and provides real-time updates on client status. Data from the participant’s app will be pushed to the clinical portal upon completion of any user interaction or when the device is charging. Clinicians will only have access to their assigned clients. They will be able to view information such as stress/craving trends, journal responses, and geolocation which can help inform their treatment plan. Treatment providers will have at-a-glance access to a home dashboard for their patient list and pending alerts, and individual patient-level data (recorded stress and craving events, extent and content of user interaction, participant use and compliance, sleep, activity, and visualizations to place stress and craving events in context). The RAE clinical portal will also generate patient reports and summaries in various formats that can be integrated into the electronic medical record for harmonization with the reminder of their clinical record.

## METHODS

### Specific Aims

Our proposal has two specific aims. Our first aim is to deploy and optimize the RAE system in a population of individuals in treatment for SUD. By integrating the RAE system into an active treatment program, we will evaluate the sensitivity/specificity of the detection algorithms in natural environments and collect data on usability from both participants and clinicians. Data from Aim 1 will be used to optimize the accuracy, usability, and compliance of the RAE system. We anticipate that data collection for Aim 1 will take 12 months, and that preliminary data analysis will be complete by June 2021. Prior to advancing to Aim 2 we will achieve preset milestones for accuracy of the stress detection algorithm and user compliance with the RAE system. Our second aim is to demonstrate the impact of the RAE system on outcomes in SUD. After optimization of the RAE system in Aim 1, we will deploy the technology in a multi-site, randomized controlled trial (RCT) to measure impact on relapse rates, engagement/retention in treatment programs, and psychosocial factors.

### Aim 1: Observational Trial for Accuracy and Usability

This study is approved by the institutional review board at the University of Massachusetts Medical School. A convenience sample of *N* = 50 participants will be recruited through multiple outpatient SUD treatment programs across the US. Five recruitment sites have been selected to increase gender, socioeconomic, and ethnic diversity in the sample. Sites vary by client payer mix (predominantly private pay vs predominantly public insured), and geographic region (two sites in the northeast, one site in the southeast, one site in the Midwest and one site in the west). All sites include both MOUD and non-MOUD treatment options. We will aim to recruit approximately 50% female participants, and a minimum of 20% African American/Black and 20% Latinx participants.

Eligible participants will be at least 18 years old, enrolled in an outpatient SUD treatment program, have an iOS or Android capable smart phone, able to communicate in English and able to provide informed consent. We will exclude individuals with a limitation of the non-dominant upper extremity (such as amputation or fracture). During the enrollment visit, study staff will obtain informed consent, and will assist the participant with downloading the RAE app onto their phone and pairing the sensor with the app. Participants will be given a brief (10 min) tutorial on app usage, including logging stresses and cravings, adding contacts, and inputting journal entries.

During the 30-day active study period, participants will be asked to wear the sensor at all times except when charging (typically required for 2–3 h every 5–7 days), as the sensor can safely be worn during normal activities including showering. Participants will be instructed to keep the RAE app open, and to interact with the RAE application when prompted by a notification or when they perceive stress or craving that was not detected by the RAE system. Study staff will conduct check in calls with participants every ten days in order to ensure compliance with the protocol, troubleshoot any technical issues, and to gather acceptability and usability data. Participants will also have access to study staff via phone, test and email during business hours for any troubleshooting or technical difficult. At the end of the active study protocol, study staff will conduct a semi-structured interview to gather feedback on user experiences.

### Data Collection

#### Quantitative data collection

Biometric (physiologic) data will be captured through the wearable sensor, streamed through the RAE app, and stored in a secure Amazon Web Services (AWS) server. Biometric data at key events will be analyzed, including: false positives (defined as a sensor detected event where the user response “No Stress/No Craving/Neutral” when prompted), true positives (defined as a sensor detected event that the user responds “Yes” to the prompted screen upon detection of stress or craving) and false negatives (defined user manually indicates stress or craving that has not been detected by the sensor). Demographic and historical data (including substance use history, medical history and psychiatric history) will be obtained at baseline. Throughout the study period, we will collect data on system compliance (number of hours per day of active data collection), number of interactions with the device (both prompted by device detection and unprompted), number of events detected, and degree of user interaction with the app (choice to interact or ignore, choice of interaction type, etc.).

Data on treatment related outcomes will be collected both through self-report and provider report at 30 days, and three, six, and twelve month follow ups. These include relapse to use of the drug for which they are receiving treatment (either via self-report or positive routine drug screen per treatment protocol), retention in treatment program, emergency department visits, and need for inpatient admission.

Assessments of functional status (with respect to education, employment, and legal issues), mental health status and positive changes in social values and networks will also be evaluated at multiple timepoints. To assess the impact of RAE on important psychosocial factors, we will utilize standardized assessment tools to obtain a more comprehensive picture of the participants’ overall health at baseline, after the 30-day active study period, and at three, six and twelve month follow up phone calls. These tools will include: The World Health Organization Quality of Life (WHOQOL) Brief tool [36–38], the Behavior and Symptom Identification Scale (BASIS-24) [39,40], and the Brief Addiction Monitor (BAM) [41]. The WHOQOL Brief is a 26-item questionnaire that focuses on individual perceptions on satisfaction with life related to physical health, psychological status, social relationships and environment. The WHOQOL Brief which has been well validated across a variety of medical and psychiatric conditions. The BASIS-24 is a validated self-report measure of mental health symptoms, relationships and ability to function in domains related to those symptoms. The BAM is a validated, 17 item self-report measure that to assess substance use disorder treatment progress and evaluates recent substance use, risk of return to substance use, and recovery protective factors.

#### Qualitative data (exit interviews and focus groups)

Data on the usability, acceptability, and overall perceptions of the RAE system will be gathered by semi-structured exit interviews with individual RAE participants. Topic areas will include perception of stress and craving, usability and acceptability of the RAE system, user experience, degree to which interactions were bothersome, degree to which continuous physiologic monitoring and/or RAE interactions affected behavior, and future implications and uses. Two focus groups will be conducted with 6–10 SUD treatment clinicians from the recruitment sites; once when 50% target enrollment is reached then again once 100% target individual enrollment is reached. We will specifically aim to engage treatment providers who have been involved with study participants, however will also invite treatment providers who have never seen RAE as well. During focus groups, the clinicians will receive demonstrations on the study devices, RAE clinical portal, and RAE mobile application. Topic areas will include current treatment provider needs amendable to digital interventions, initial impressions of the mobile app/clinician portal, RAE workflow integration, and barriers/facilitators to RAE implementation into practice. Interviews and focus groups discussions will be audio recorded, then transcribed verbatim.

### Data Analysis

#### Machine learning of biometric data, evaluation of participant characteristics, and compliance

A machine-learning framework will be developed with features derived from sensor accelerometer as well as heart rate data using Hilbert transform and wavelet transform approaches. The features include shape and scale of the amplitude distribution of the accelerometer data along with nonlinear features of other available signals [28]. The algorithm will initially be evaluated using 10-fold cross validation, and subsequently be split into training and test data sets as sufficient data become available. The machine learning paradigm will provide us with a classifier that can distinguish stress and cravings from non-stress conditions. Models will be evaluated by standardized metrics of sensitivity, specificity and Area Under the Curve (AUC) for the Receiver Operating Characteristics (ROC) curve. Our goal is to improve upon our base algorithm an AUC value of 0.8 or above. We will improve the performance of these standard classifiers with features based on the nonlinear characteristics of the data and if we cannot achieve the desired performance, we will advance to deep learning methods. Individual participant and treatment characteristics associated with detection accuracy and system compliance will be explored to identify both at-risk individuals and characteristics that would indicate need for a tailored approach. Specifically, we will evaluate the effect of gender, substance of choice, medications for opioid use disorder (MOUD, i.e., methadone, buprenorphine, and/or naltrexone), and age on digital biomarkers and compliance.

Overall rates of compliance with the system will be calculated, including the hours per day of use (based on biometric data capture) and the frequency and extent to which participants respond to the RAE system prompts. In addition, Relative Subjective Count (RSC), the quotient of the participant’s estimate of the number of times RAE requested and annotation by the actual number of interactions, will be calculated [42]. This measure indirectly assesses the patient’s overall satisfaction with a technological system, and serves as a segregate measure of usability and acceptability. Low RSC (<1.0) correlates with increased participant satisfaction, while high RSC (>1.0) reflects poor usability of a technology.

#### Qualitative data analysis

Thematic analysis will be applied to exit interview and focus group qualitative data. The coding structure will initially be developed based on deductive codes from the interview guide, and then inductive codes will be added after review of the interview transcripts. Once the coding scheme is developed, each transcript will be double coded by two investigators independently. After the transcripts are compared to ensure comprehensiveness of coding, the agreed-upon codes will be entered into qualitative data analysis software (NVivo, QRS International, Burlington, MA, USA). Resultant summaries arising from each code will be used to adapt and refine RAE components, interaction parameters, and other technology features.

### Aim 2: Randomized Controlled Trial

After optimization of the RAE system in Aim 1, we will deploy the technology in a multi-site randomized controlled trial to measure impact on relapse rates, engagement/retention in treatment programs, and functioning/quality of life. Data from this Aim 2 pivotal study will be used to apply for FDA approval for the RAE system. During the RCT, the RAE system will be deployed as a part of SUD treatment programs at multiple outpatient sites nationwide. The goal is for RAE to be a tool that clinicians and patients can use to augment their treatment program; as such, in this phase, participants (clinician-patient dyads) will be provided with electronic instructional materials on the app, clinical portal and device, but study staff will otherwise not interfere with routine treatment protocols.

We will recruit *N* = 300 participants across the same five study sites sampled in Aim 1. The exact expected effect size of the intervention is difficult to calculate given the wide range of reported values for relapse rates for individuals in recovery from SUD. Using the NIH reported rate of relapse (40–60%), we conservatively estimate a mean rate of relapse in the control population is 50%. A sample size of 264 participants (study wide) would then allow us to detect a 10% decrease in relapse rate in the RAE group with 90% power and 5% alpha level. We have inflated the sample size by approximately 15% to account for attrition and loss to follow up.

Inclusion and exclusion criteria will be identical to Aim 1 (described above). Participants will be randomized in a 1:1 fashion using a computer-generated stratified randomization sequence to RAE plus standard treatment vs standard treatment alone (control). Participants in control group will receive a Garmin Vivosmart 4 wearable sensor to control for any effect of general health and wellness monitoring with a wearable, but will not have access to other RAE components. Given that the use of the RAE system is physically obvious, there will be no blinding with the exception of the standardized assessments for quality of life and functional status, and follow up data collection tools (described above): These tools will be administered and scored by blinded study staff to reduce the opportunity for bias in these outcome measures.

The primary outcome for the RCT will be relapse to substance use at 3, 6, and 12 months defined as (1) self-reported use of the substance they are in treatment for, (2) a positive urine drug screen for the same substance or (3) provider confirmed return to use. The secondary outcomes will be: retention in treatment program and change in baseline scores on the WHOQOL, BASIS-24 and BAM scales at 3, 6, and 12 months.

### Data Analysis

We will collect measures identical to those outlined in Aim 1, and will consult machine learning analysis and qualitative analysis to refine the detection algorithms and the quality of the intervention. The primary analysis for Aim 2 will be an intention-to- treat analysis to evaluate the impact of RAE on relapse to substance use, however we will also perform a per-protocol analysis. We will compare overall rates of relapse, and will determine relative risk of relapse in the RAE group vs control group. We use logistic regression models to adjust for treatment center, SUD subtype, treatment type (MOUD vs non-MOUD), age and gender. We will also compare change in scores of psychosocial functioning assessments over time in RAE users vs controls, using time series analysis and adjusting for the same for covariates.

### Limitations

We acknowledge several limitations inherent in our study design and of the RAE system. First, the validation of the system using self-reported symptoms (craving/stress) as the gold standard presents opportunities for misclassification; for example, if a user is experiencing a subtle stress event that is not detected by RAE and that they do not recognize and acknowledge, this represents a missed false negative. However, as the individual uses RAE for a longer period of time, the goal is to use machine learning to improve detection of users’ stress and cravings to minimize false negatives. Second, the current protocol aims to evaluate the efficacy of RAE for a 30 day use period, although it is unknown what the optimal dose/duration of the intervention is. Future studies will need to address the impact of length and timing (with respect to the initiation of treatment) of RAE on its effectiveness. Third, RAE currently relies on and intact support system; challenges experienced by users who lack support structure will need to be addresses separately.

As with all mHealth interventions, barriers to user engagement, specifically privacy and trust issues from the end user perspective, could limit efficacy if not addressed. From a technical perspective, RAE provides HIPPA compliant data management in a secure environment, which is expected to alleviate concerns from treatment providers. But for individuals in recovery from SUD, regulatory compliance standards alone may not be sufficient to alleviate privacy concerns. We believe that RAE will be more effective as an accountability tool that individuals in recovery can use to partner with providers, rather than mandatory monitor or “big brother.” To set this tone (and to build trust), we have implemented several strategies. The system is an opt-in technology; if a user wants to halt data collection, they can simply remove the sensor and/or turn off the app. Non-compliance will be apparent to their treatment provider and will raise questions, but is always an option. The GPS location feature, which is arguably the most invasive, can also be turned off at the client/treating providers discretion. Ongoing and future work will need to continuously address privacy and trust issues as a central component of the user experience and design to optimize engagement and, in turn, the opportunity for efficacy.

## DISCUSSION

There is an urgent need for novel strategies to combat the growing SUD epidemic. mHealth technologies provide a means for individuals in recovery to consistently track physiologic and behavioral markers of risk while allowing the opportunity to for just-in time adaptive interventions and support. The development of the RAE system provides a novel tool for individuals with SUD, the clinicians who care for them, and the scientific community/mHealth research space.

For the individual with SUD in recovery, the potential benefits of RAE include automated detection of high-risk events, de-escalation tools, contact with support systems, and accountability tracking. The ultimate goal is for these tools to provide the user valuable insight into their own behavior, opportunities for self-reflection, and a sense of connectedness and support.

For SUD treatment clinicians, the potential in the RAE system lies in the opportunity to monitor patient risk levels and visualize data to understand trends and triggers, many of which may not be readily apparent to the user or clinician. This ability of clinicians to use objective measures of risk to tailor interventions for individuals in recovery could prevent relapse, improve rates of sustained recovery, and save lives compared to current treatment paradigms. Mobile health tools such as RAE are a particularly attractive way for clinicians to connect with patients in the current challenges of COVID-19 related social distancing protocols, making study of this approach particularly timely [43,44].

For the general scientific community and knowledge base, the refinement of digital biomarkers of craving and stress developed through RAE have the potential to enhance our understanding of the biological underpinnings of these phenomenon, and of the mechanics of addiction, facilitating the provision of personalized medicine. If successful, outcomes from this work would lay the groundwork for other real-time, automated behavioral interventions that are triggered by digital biomarkers. This translates to a personalized intervention approach, an area that behavioral research experts have identified as a critical next step for the field of drug abuse treatment. This novel digital intervention provides a plausibly low-cost mechanism to enhance the success of SUD treatment. Furthermore, RAE can also be tested as a stress management option for other high-risk populations with and without SUD, such as healthcare providers, justice involved individuals, and those with other mental health diagnoses.

## ABBREVIATIONS

SUD: Substance use disorder
mHealth: Mobile health
AUD: Alcohol Use Disorder
EMA: ecological momentary assessment
DBT: dialectical behavioral therapy
MOUD: Medications for Opioid Use Disorder
RCT: Randomized controlled trial
